# Silicon, by promoting a homeostatic balance of C:N:P and nutrient use efficiency, attenuates K deficiency, favoring sustainable bean cultivation

**DOI:** 10.1186/s12870-023-04236-5

**Published:** 2023-04-24

**Authors:** Milton G. Costa, Renato de M. Prado, Marcilene M. Santos Sarah, Jonas P. Souza Júnior, Antonia Erica S. de Souza

**Affiliations:** Faculty of Agricultural and Veterinarian Sciences. Department of Agricultural Production Sciences, São Paulo State University (UNESP), Jaboticabal, Via de Acesso Prof. Paulo Donato Castellane, São Paulo, 14884900 Brazil

**Keywords:** *Phaseolus vulgaris* L., Nutritional stress, Beneficial element, K sufficiency, Nutritional stoichiometric ratios

## Abstract

**Background:**

In many regions of the world, K is being depleted from soils due to agricultural intensification a lack of accessibility, and the high cost of K. Thus, there is an urgent need for a sustainable strategy for crops in this environment. Si is an option for mitigating stress due to nutritional deficiency. However, the underlying effects of Si in mitigating K deficiency C:N:P homeostasis still remains unknown for bean plants. This is a species of great worldwide importance. Thus, this study aims to evaluate whether i) K deficiency modifies the homeostatic balance of C, N and P, and, if so, ii) Si supply can reduce damage caused to nutritional stoichiometry, nutrient use efficiency, and production of dry mass in bean plants.

**Results:**

K deficiency caused a reduction in the stoichiometric ratios C:N, C:P, and P:Si in shoots and C:N, C:P, C:Si, N:Si, and P:Si in roots, resulting in a decrease in K content and use efficiency and reducing biomass production. The application of Si in K-deficient plants modified the ratios C:N, C:Si, N:P, N:Si, and P:Si in shoots and C:N, C:P, C:Si, N:Si, N:P, and P:Si in roots, increasing the K content and efficiency, reducing the loss of biomass. In bean plants with K sufficiency, Si also changed the stoichiometric ratios C:N, C:P, C:Si, N:P, N:Si, and P:Si in shoots and C:N, C:Si, N:Si, and P:Si in roots, increasing K content only in roots and the use efficiency of C and P in shoots and C, N, and P in roots, increasing the biomass production only in roots.

**Conclusion:**

K deficiency causes damage to the C:N:P homeostatic balance, reducing the efficiency of nutrient use and biomass production. However, Si is a viable alternative to attenuate these nutritional damages, favoring bean growth. The future perspective is that the use of Si in agriculture in underdeveloped economies with restrictions on the use of K will constitute a sustainable strategy to increase food security.

## Background

Climate change increases global temperatures. Some regions experience droughts and immediately thereafter a sudden burst of excessive rainfalls [1]. Excessive rainfalls result in further destructive and dangerous natural hazards, that is, floods, which cause serious damage to life and soils every year. An increase in crop flooding area from 10 to 20% by 2080 is expected [2]. In soils, excess rainfall promotes leaching of cations, especially K, decreasing its availability in the soil solution [3]. This is more serious in weathered soils containing source materials of low nutrient availability. Also, soils in some regions of the southern hemisphere are currently running out of K due to the expansion and intensification of agriculture and a lack of access to potassium [4]. It is estimated that the amount of potassium required by agricultural crops is ten times greater than the amount extracted annually by the fertilizer industry [5], resulting in a deficiency of this nutrient in a large part of crops. On the other hand, drought decreases the movement of ions in the soil, including K^+^, reducing its uptake by plants [6]. Therefore, it is evident that climate change may accentuate problems of potassium deficiency in crops. Losses by K deficiency are more drastic in K-demanding species, such as beans, a crop of global importance, as it causes damage to the sustainability of the crop [7] and leads to food insecurity.

Thus, K deficiency causes well-known physiological damages [8, 9]. Examples are osmotic imbalance and losses in photosynthetic rates due to low water use efficiency [10, 11]. In addition, K activates dozens of enzymes in different metabolic pathways in plants [12–15], with an emphasis on N metabolism [16]. This consequently reduces plant growth. However, little is known about the damage caused by K deficiency to the stoichiometric homeostasis of C:N:P and its effects on nutritional efficiency, that is, on the plant's ability to use nutrients in physiological and biochemical processes that are vital for conversion into dry mass. There is recent strong evidence for other species that stresses such as saline [17] and water deficit [18, 19], associated with increased temperature [20], are responsible for elemental imbalances in stoichiometry enough to decrease the nutritional efficiency of crops and plant growth This may occur with K deficiency, but there is not enough evidence of it. Additionally, potassium is also known to reduce the impacts of stressful conditions in plants. Its deficiency is severe, as it reduces plant tolerance to other stresses and consequently leads to yield losses [21].

An innovative and sustainable strategy to mitigate stress caused by K deficiency can be the use of Si. This is because Si is known to mitigate different abiotic stresses, including in legumes [22–25]. Little is known about the potential of Si to attenuate the biological damage of K deficiency in bean plants. Recently, researchers discovered that Si improves the uptake of K in soybean plants, improving the response of plants under saline stress [26]. There is only one study reporting the benefits of Si to the photosynthetic apparatus [27], a fact also observed for maize plants [28], basil plants [29], peanut (*Arachis hypogaea*) [24], and barley (*Hordeum vulgare* L.) [30, 31]. However, it is necessary to advance in the research on elementary stoichiometric homeostasis for a better understanding of the effects of Si on this nutritional balance for a possible neutralization of damages arising from K deficiency.

Studies have shown the role of Si in replacing C in organic compounds that make up the cell wall of plants, especially lignin and cellulose [32]. Recent research indicates that Si uptake can lead to a new homeostatic balance of C:N:P, as observed in different species such as *Sorghum bicolor* (L.) Moenchand, *Helianthus annuus* L. [17, 33], *Chenopodium quinoa* Willd. [34], *Triticum aestivum* L. [35], *Panicum maximum* L. [36], *Phragmites australis* [37], *Medicago sativa* [38], and *Saccharum officinarum* L. [19, 39–43]. In this scenario, there are indications that this new elemental homeostasis of C, N, and P promoted by Si plays a role in minimizing biomass losses in plants of different species under stress conditions [39–41] or enhancing plant growth without stress [41, 44]. However, this is not yet studied in bean plants.

In this context, studies are needed to understand the biological damages that K deficiency causes to the homeostatic balance of C:N:P and the role of Si in attenuating nutritional deficiency. For this, it is relevant to test the following hypotheses: (i) K deficiency can cause damage to the homeostatic balance of C:N:P, reducing the use efficiency of these nutrients, (ii) Si can reverse these nutritional damages and improve the nutritional efficiency and growth of K-deficient bean plants, which may mean a possible benefit to agriculture in underdeveloped economies with restricted use of K, or (iii) Si could improve nutritional balance and efficiency in a level that is enough to increase dry mass production of common beans cultivated under K sufficiency, meaning a possible use of Si in agriculture in developed economies with no nutritional restrictions.

This research aims to evaluate whether K deficiency changes the stoichiometric ratio C:N:P of bean plants and whether the supply of Si can modify the homeostatic balance of C:N:P, attenuating the damage caused by K nutritional deficiency and increasing the use efficiency of nutrients and the production of dry mass of bean plants with deficiency and sufficiency of K.

If these hypotheses above are true, they should reveal one more benefit of Si in mitigating stresses such as potassium deficiency in bean plants resulting from the improvement in the nutritional efficiency of C, N, and P. Thus, this research may pave the way for further studies on different species. It may open ways to evaluate the role of Si in the stoichiometric homeostasis of C, N, and P, which are vital structural nutrients for plant metabolism, in order to better explain the optimal performance of nutritionally deficient crops that received Si, which is still little discussed in studies on Si.

## Results

### Si, C, N, and P concentrations

**Fig. 1:**
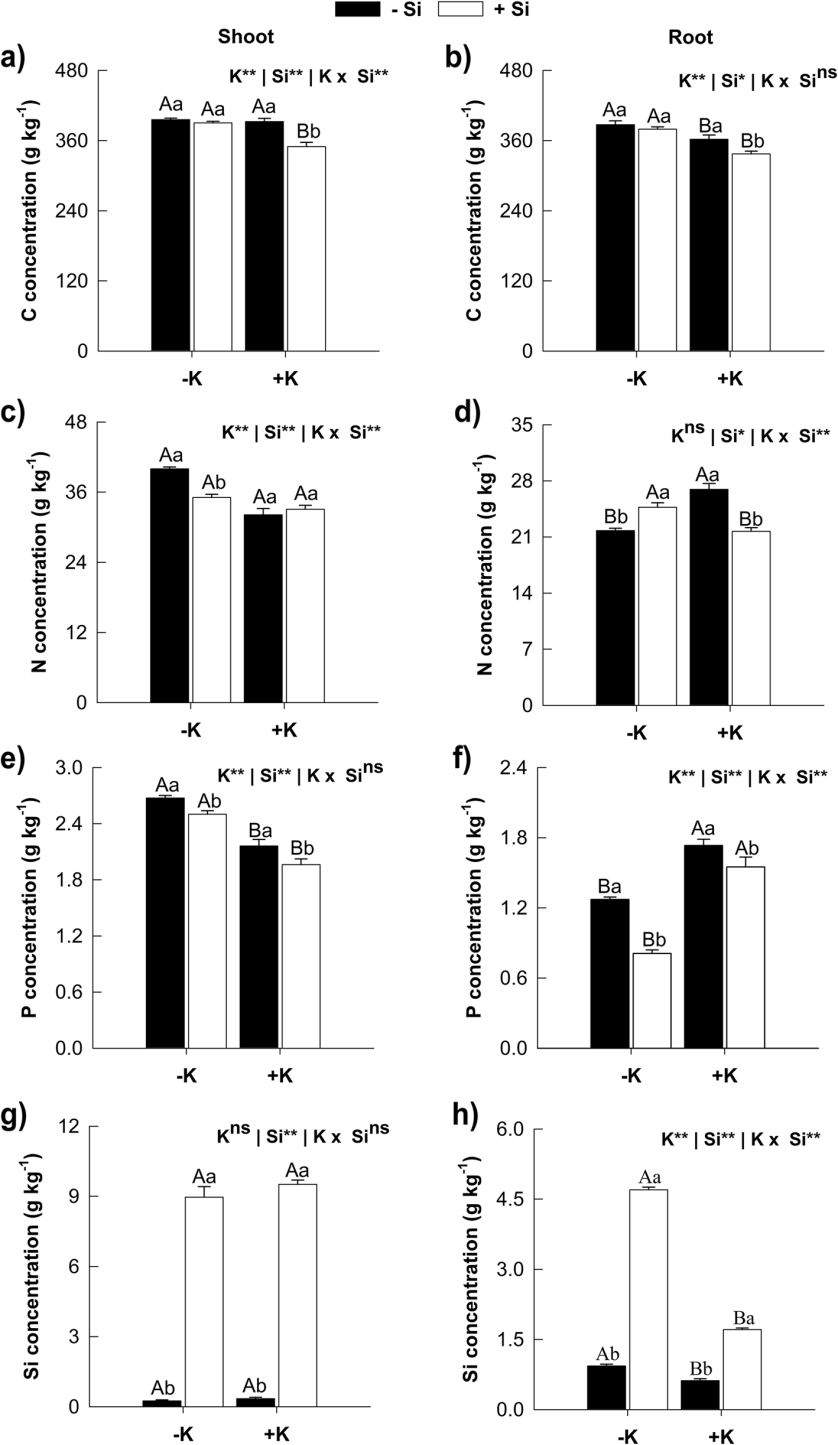
C (**a**, **b**), N (**c**, **d**), P (**e**, **f**), and Si (**g**, **h**) concentration in leaves and roots of bean plants cultivated under deficiency (-K) and sufficiency (+ K) of K in the absence (-Si) and presence of supply of Si (via nutrient solution). Different uppercase letters indicate differences in K supply (deficiency or sufficiency) and different lowercase letters indicate differences in Si application (absence and presence of Si) by Tukey test (*p* < 0.05). Error bars indicate mean standard error (*n* = 5)

K deficiency decreased the concentration of Si and C in roots (Fig. 1b and h), decreased the concentration of P in shoots (Fig. 1e) and increased the concentration of N and P in roots in the absence of application of the beneficial element (Fig. 1d and f). The application of Si to bean plants with K deficiency led to an increase in the concentration of Si in shoots (Fig. 1g) and N, P, and Si in roots (Fig. 1b, d and h). There was also a decrease in the concentrations of N and P in shoots (Fig. 1c and e). Regarding the nutritional sufficiency of K, the application of Si decreased the concentration of C in shoots (Fig. 1a) and C, N, and P in roots (Fig. 1b, d and f) and increased the concentration of Si in shoots and roots (Fig. 1g and h).

### Homeostatic balance of C, N, P, and Si

**Fig. 2:**
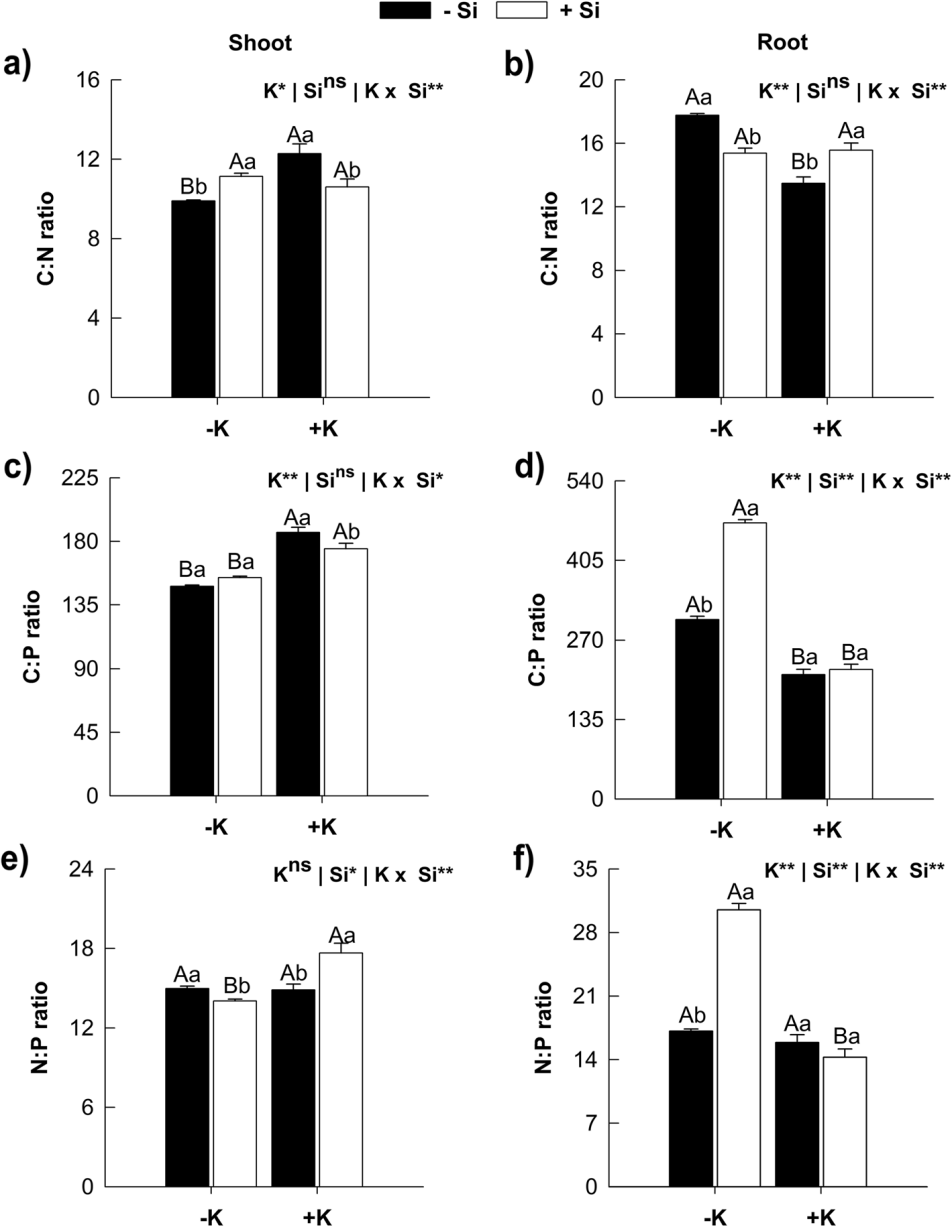
Stoichiometric ratios C:N (**a**, **b**), C:P (**c**, **d**), and N:P (**e**, **f**) in leaves and roots of bean plants cultivated under deficiency (-K) and sufficiency (+ K) of K in the absence (-Si) and presence of supply of Si (via nutrient solution). Different uppercase letters indicate differences in K supply (deficiency or sufficiency) and different lowercase letters indicate differences in Si application (absence and presence of Si) by Tukey test (*p* < 0.05). Error bars indicate mean standard error (*n* = 5)

**Fig. 3:**
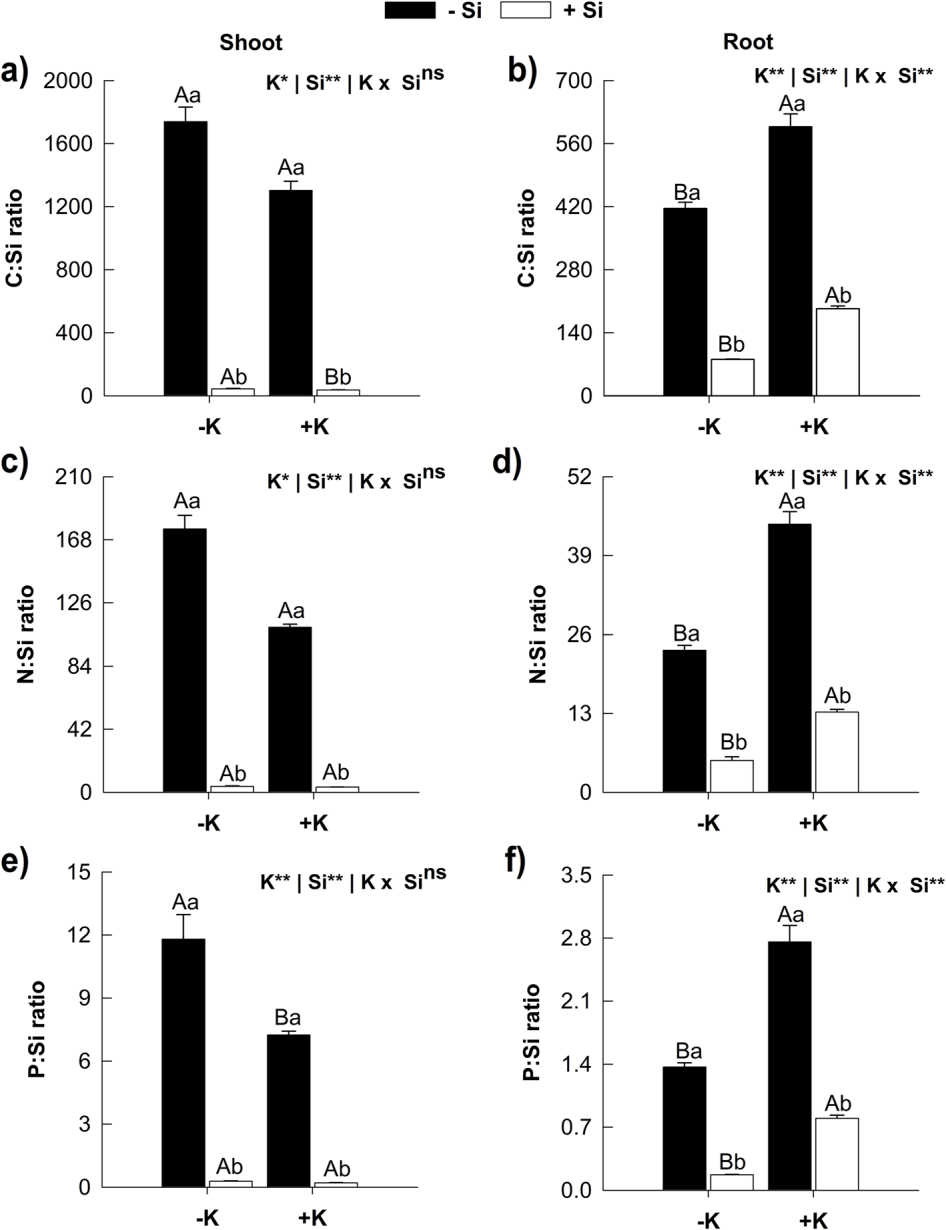
Stoichiometric ratios C:Si (**a**, **b**), N:Si (**c**, **d**), and P:Si (**e**, **f**) in leaves and roots of bean plants cultivated under deficiency (-K) and sufficiency (+ K) of K in the absence (-Si) and presence of supply of Si (via nutrient solution). Different uppercase letters indicate differences in K supply (deficiency or sufficiency) and different lowercase letters indicate differences in Si application (absence and presence of Si) by Tukey test (*p* < 0.05). Error bars indicate mean standard error (*n* = 5)

The stoichiometric ratios C:N and C:P in shoots (Fig. 2a and c) and C:Si, N:Si, and P:Si in roots (Fig. 3b, d and 3f) decreased, and the stoichiometric ratios P:Si in shoots (Fig. 3e) and C:N and C:P in roots (Fig. 2b and d) increased due to K deficiency in bean plants without Si application. However, there was no change in N:P and N:Si ratios in shoots (2e and 3c) and N:P in roots (Fig. 2f). In K-deficient plants, the supply of Si increased the stoichiometric ratios of C:N in shoots (Fig. 2a) and C:P and N:P in roots (Fig. 2d and f), but it decreased the ratios N:P, C:Si, N:Si, and P:Si in shoots (Figs. 2e, 3a, c and e) and C:N, C:Si, N:Si and P:Si in roots of bean plants (Figs. 2b, 3b, d and f). In plants with K sufficiency, there was a reduction in the stoichiometric ratios C:N, C:P, C:Si, N:Si, and P:Si in shoots (Figs. 2a, c, 3a, c and e) and C: Si, N:Si and P:Si in roots (Fig. 3b, d and f) and an increase in the stoichiometric ratios N:P (shoots) and C:N (roots) (Fig. 2e and b).

### C, N, P, and Si contents

**Fig. 4:**
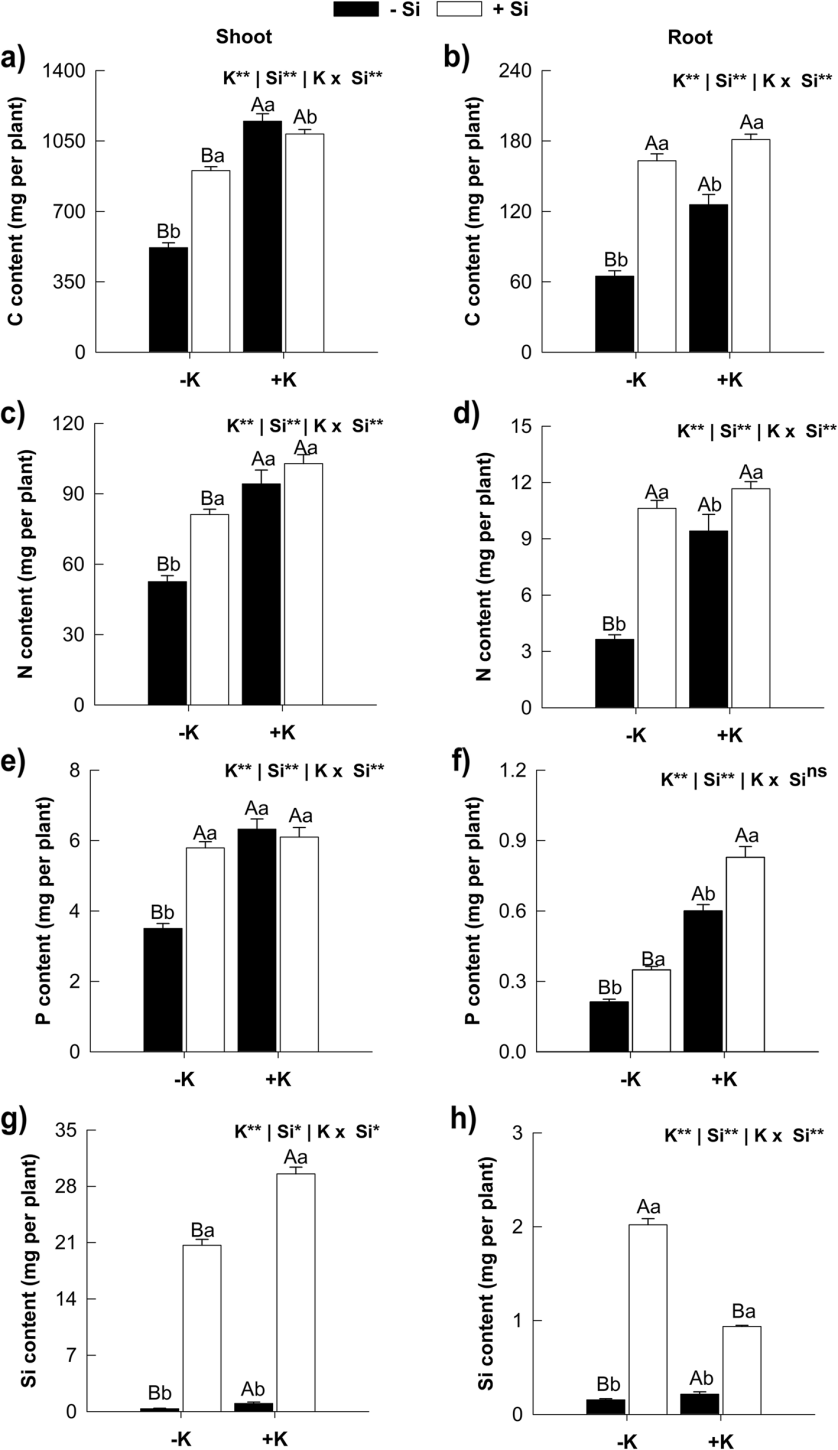
C (**a**, **b**), N (**c**, **d**), P (**e**, **f**), and Si (**g**, **h**) content in leaves and roots of bean plants cultivated under deficiency (-K) and sufficiency (+ K) of K in the absence (-Si) and presence of supply of Si (via nutrient solution). Different uppercase letters indicate differences in K supply (deficiency or sufficiency) and different lowercase letters indicate differences in Si application (absence and presence of Si) by Tukey test (*p* < 0.05). Error bars indicate mean standard error (*n* = 5)

K deficiency caused a reduction in C, N, P, and Si content in shoots and roots in the absence of Si supply in bean plants (Fig. 4). The application of Si increased the contents of C, N, P, and Si in shoots and roots in plants under K deficiency, while in bean plants under K sufficiency there was an increase in Si content in shoots (Fig. 4g) and of C, N, P, and Si in roots (Fig. 4b, d, f and h).

### C, N, and P use efficiency and biomass production

**Fig. 5:**
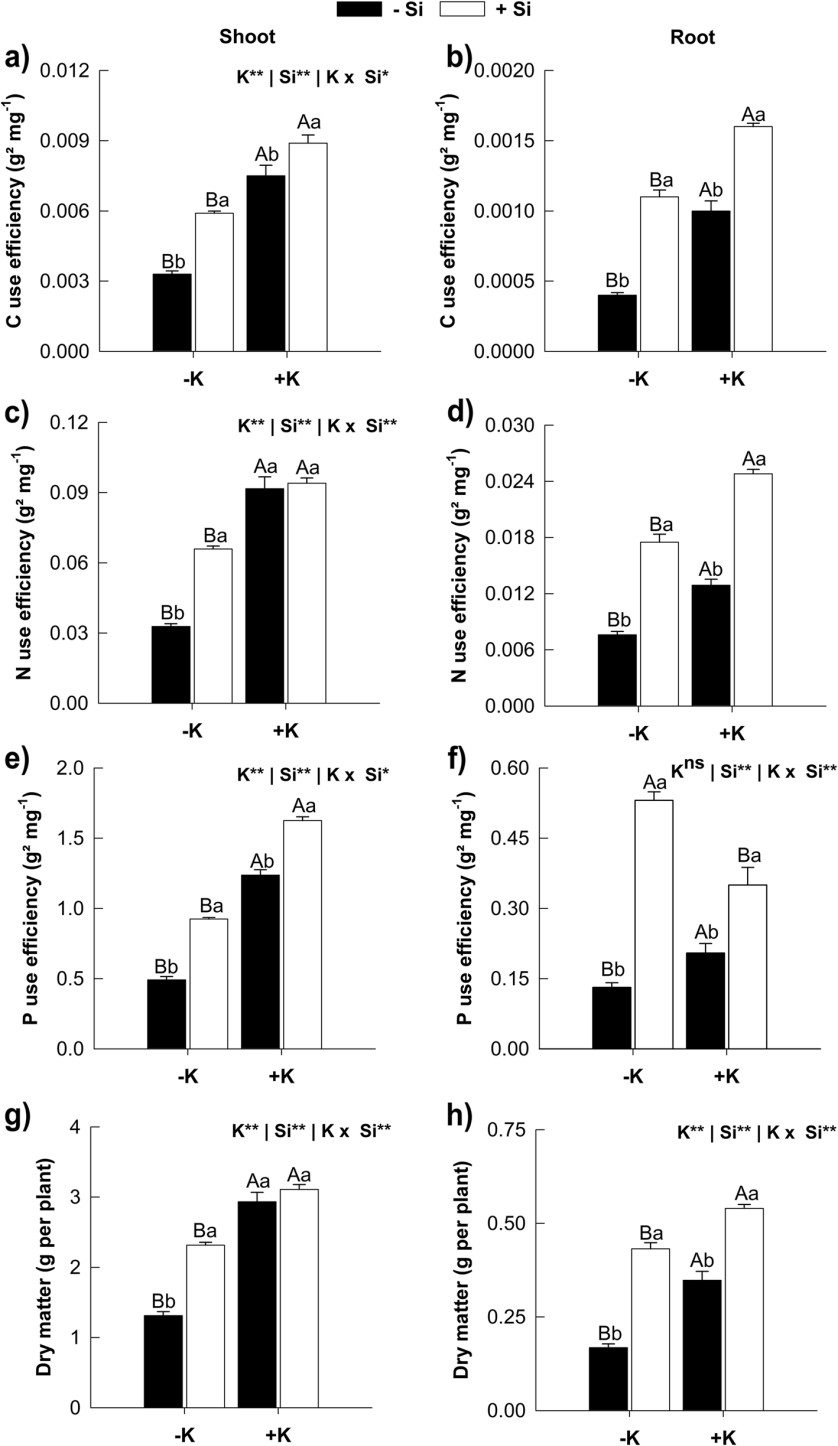
C (**a**, **b**), N (**c**, **d**), and P (**e**, **f**) use efficiency and production of dry mass (**g**, **h**) in leaves and roots of bean plants cultivated under deficiency (-K) and sufficiency (+ K) of K in the absence (-Si) and presence of supply of Si (via nutrient solution). Different uppercase letters indicate differences in K supply (deficiency or sufficiency) and different lowercase letters indicate differences in Si application (absence and presence of Si) by Tukey test (*p* < 0.05). Error bars indicate mean standard error (*n* = 5)

C, N, and P use efficiency in shoots and roots of bean plants decreased in plants under K deficiency in relation to K sufficiency and in the absence of Si supply (Fig. 5). The supply of Si increased the use efficiency of C, N, and P in shoots and roots of K-deficient plants (Fig. 5). In plants with K sufficiency, the addition of Si to the nutrient solution also increased the C and P use efficiency in shoots (Fig. 5a and e) and C, N, and P in roots (Fig. 5b, d and f).

K deficiency in common bean plants in the absence of Si supply caused a reduction in the production of shoot and root dry matter (Fig. 5g and h). The supply of Si increased the production of shoot and root mass in K-deficient plants, while in plants with K sufficiency there was only an increase in the dry mass of roots (Fig. 5h).

### Hierarchical cluster analysis

**Fig. 6:**
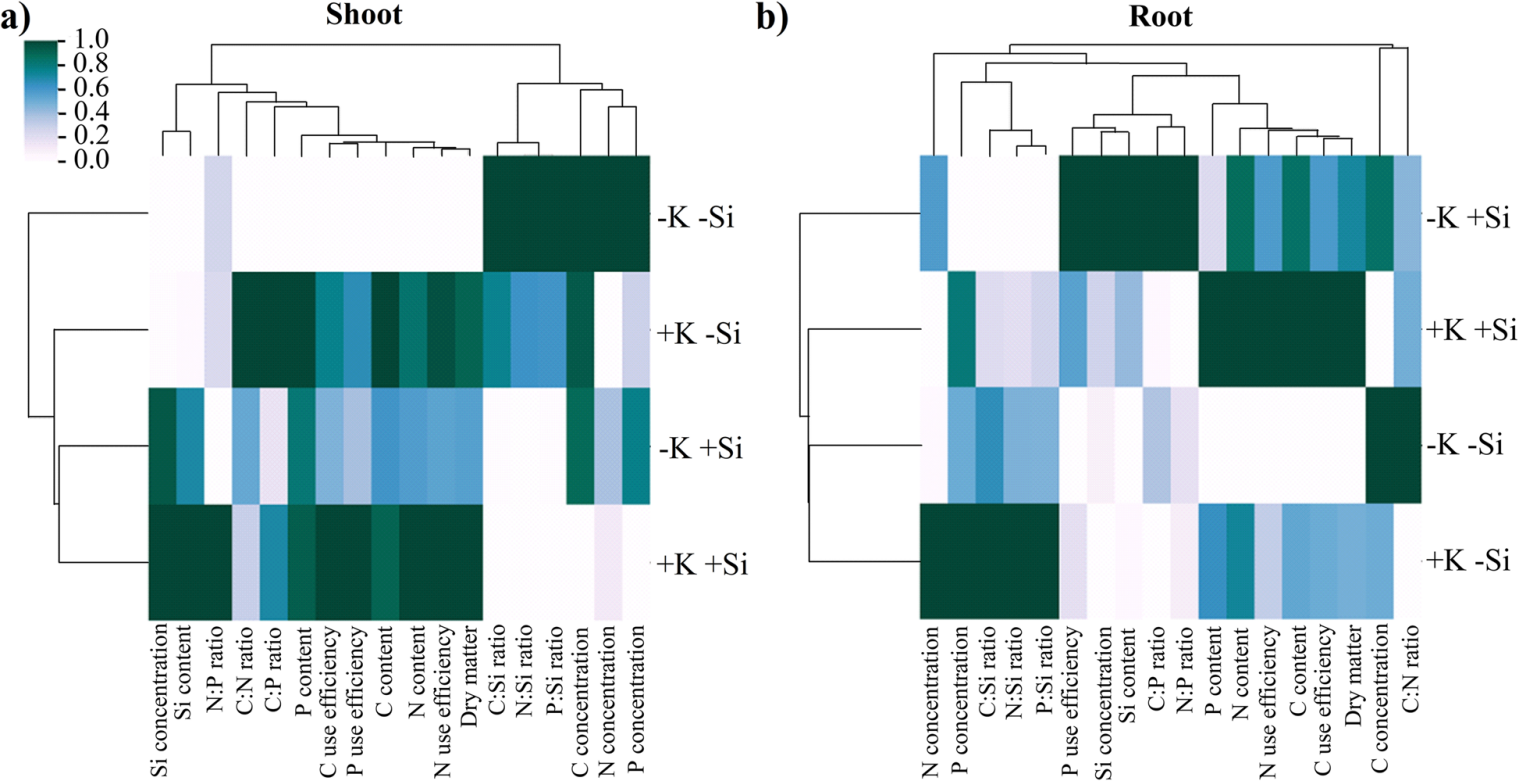
Correlogram of the stoichiometric ratio C:N:P, C, N and P use efficiency, dry mass production (**a**, **b**), and hierarchical cluster heat map of independent variables in leaves and roots of bean plants cultivated under deficiency (-K) and sufficiency (+ K) of K in the absence (-Si) and presence of supply of Si (via nutrient solution)

The hierarchical cluster analysis of shoots showed that there was a greater dissimilarity in the absence of Si supply in K-deficient plants in treatments that received application of Si and in the treatment with K sufficiency in the absence of Si (Fig. 6a). In the analysis of hierarchical clustering in roots, the greatest dissimilarity in the Si supply condition in K-deficient plants occurred in treatments that did not receive Si application and in the treatment with K sufficiency and application of Si (Fig. 6b).

For shoots, the hierarchical cluster analysis showed a greater similarity of variables of the stoichiometric ratios C:N and C:Si and concentrations of C, N, and P in the absence of supply of Si to plants deficient in K, while Si concentration was associated with treatments that received Si application (Fig. 6a). The use efficiency of C, N, and P, the stoichiometric ratio N:P, and the contents of N and Si were associated with the treatment that received a supply of Si under K sufficiency. The increase in the stoichiometric ratio C:N and C:P and in the contents of C and P were associated with treatments with absence of Si supply under K sufficiency (Fig. 6a).

In the roots of bean plants, there was a greater similarity of P use efficiency, Si concentration and content, and the stoichiometric ratios C:P and N:P with the treatment with Si supply under K deficiency. On the other hand, there was a greater association of C concentration and the stoichiometric ratio C:N with treatments without Si supply under K deficiency (Fig. 6b). Concentrations of N and P and the stoichiometric ratio C:Si were associated with the treatment without Si supply under K sufficiency Finally, the contents of C, N and P, the use efficiency of C and N, and the dry mass production were associated with a greater similarity to the treatment with Si supply under K sufficiency (Fig. 6b).

### Principal component analysis

**Fig. 7:**
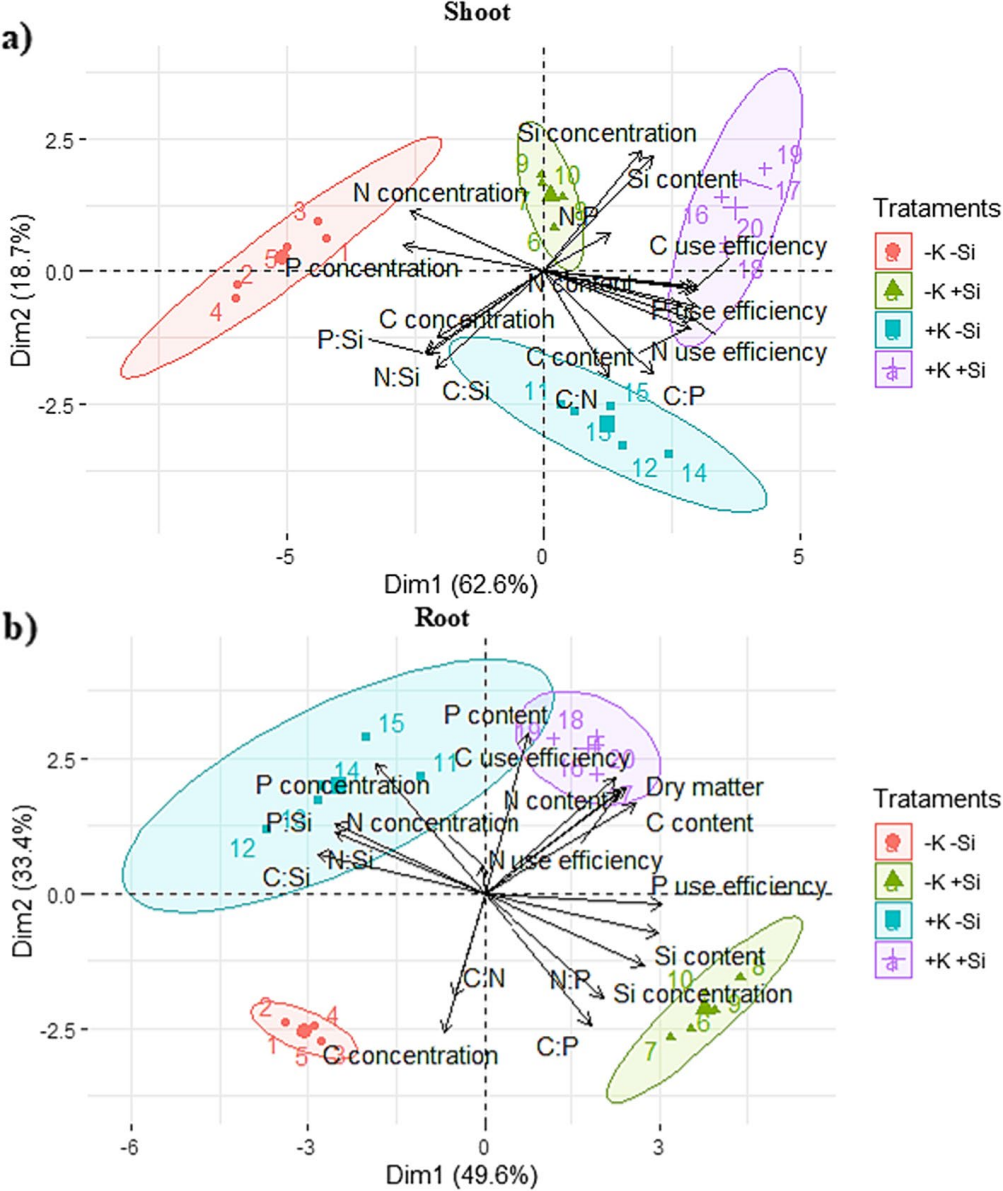
PCA of the stoichiometric ratio C:N:P, C, N and P use efficiency, dry mass production (**a**, **b**), and hierarchical cluster heat map of independent variables in leaves and roots of bean plants cultivated under deficiency (-K) and sufficiency (+ K) of K in the absence (-Si) and presence of supply of Si (via nutrient solution)

The principal component analysis (PCA) of shoots and roots of common bean plants explained 81% and 83% (principal component 1 + 2) (Fig. 7a, b). As for the PCA of shoots, dry mass, P concentration, N and P content, and C, N and P use efficiency explained the variance in PC1 and Si concentration; C:N, C:P and N:P and Si content explained the variance in PC2 (Fig. 6a); and C and N concentrations, C content and C:Si, N:Si and P:Si ratios explained the variance in PC1 and PC2 (Fig. 7a). As for the PCA of roots, dry mass, P concentration, N and P content, C, N and P use efficiency explained the variance in PC1; Si concentration and content and the ratios C:N, C: P and C:Si explained the variance in PC2; and C and N concentrations, C content, and N:P, N:Si and P:Si ratios contributed with mean values ​​to explain the variance in PC1 and PC2 (Fig. 7b).

In shoots, the PCA showed an association of increased N and P concentrations with the treatment with K deficiency in the absence of Si The increased C:N and C:P ratios was associated with treatments with K sufficiency in the absence of Si. Increased C, N and P use efficiency, C content, and dry mass were associated with treatments with K sufficiency and the presence of Si (Fig. 7a). Also, the increase in Si concentration and content and the N:P ratio was associated with treatments with the presence of Si in K deficiency and K sufficiency (Fig. 7a).

For the root system, there was an association of increased C concentration and C:N ratio, C:P and N:P ratios, P use efficiency, and P concentration and content to treatments with K deficiency and absence of Si. Si associated with treatments with K deficiency and presence of Si (Fig. 7b). The C:Si, N:Si and P:Si ratios and the N and P concentrations were associated with treatments with K sufficiency and Si absence, while C and N use efficiency, C, N and P contents, and dry mass were associated with treatments sufficient in K and the presence of Si (Fig. 7b).

## Discussion

### Silicon in the stoichiometry of C:N:P and nutritional efficiency in K-deficient beans

K deficiency in bean plants begins with a reduction in C, N, P, and Si contents in shoots and roots (Fig. 3), that is, a decrease in the uptake of these elements. This is because K deficiency can impair the water status of plants [11, 45] and decrease the activity of aquaporins, causing a reduction in the hydraulic conductance of roots [46] and resulting in less transpiration, consequently in less uptake of nutrients and physiological damage [46–48]. These processes are widely known, but it is necessary to explain whether the lack of this element causes loss of stoichiometric homeostasis of vital structural nutrients for plant metabolism, such as C, N and P. A study conducted on barley (*Hordeum vulgare* L.) aimed to evaluate the impact of potassium deficiency on the dynamics of other nutrients. The authors found changes in the dynamics of Ca, Fe, and Zn but did not observe any impact on the homeostasis of C, N, and P [30]. We argue here that the disturbance of the metabolism of these key nutrients could result in important losses in the use efficiency of these elements in plants, thus explaining the loss of dry mass in common beans.

Potassium is crucial for balancing homeostasis in plants [49]. The results show that K deficiency in bean plants results in changes in the homeostatic balance of C:N:P, decreasing the stoichiometric ratios C:N and C:P in shoots (Fig. 2a and c) and C:Si, N:Si and P:Si in roots (Fig. 3b, d and f It also increases the P:Si ratio in shoots (Fig. 3c) and C:N and C:P in roots (Fig. 2b and d).

Impairments to the homeostatic balance of C:N:P may be related to low C assimilation resulting from K deficiency [3]. This was probably due to impaired N and P metabolism, essential for an optimal C assimilation [8, 38, 50, 51].

The losses by K deficiency in bean plants causes changes in stoichiometric homeostasis of C, N and P This results in a decrease in the use efficiency of C, N and P, that is, there is a low capability of the plant to use these nutrients to ensure an optimal metabolism. It consequently decreases the plant's ability to convert dry mass in shoots and roots (Fig. 5g and h). The hierarchical cluster analysis complements this information by showing that the increase in the stoichiometric ratio C:N is the most limiting factor to the production of dry mass (Fig. 5g). This reinforces that the lower N content in relation to C points to the importance of K for N metabolism and the dry mass production of beans. Studies conducted under water deficit conditions have also shown that dry mass loss is related to a modification of the C:N:P homeostatic balance and the loss of carbon use efficiency [39, 42, 43]. The results of our work, together with the results reported in the literature, show that under stressful conditions there is damage to the plant's homeostatic balance and loss of nutrient use efficiency, such as C, N, and P.

In this context, our results show that the first hypothesis is true since K deficiency in bean plants can cause damage to the homeostatic balance of C:N:P, reducing the use efficiency of these nutrients. This shows, for the first time, that studies on the role of K in common beans should not stop in physiological evaluations involving gas exchanges or enzymatic metabolism.

The application of Si was efficient in increasing the concentration of Si in shoots and roots (Fig. 1h), indicating that there is an increase in the uptake of the beneficial element (Fig. 3g). The uptake of Si that occurs in the form of H_4_SiO_4_ is favored even under nutritional stress conditions [29], as the present study shows (Fig. 3g and h). It is a passive movement, which is common in legumes [52]. It is noteworthy that the uptake of Si, observed here by its content in roots, is greater in plants under nutritional stress, that is, deficient rather than sufficient in K. Similar results were found for peanuts, with a higher Si content in the roots of plants that were under a high K restriction [24, 53].

The Si uptake by K-deficient bean plants promoted a change in the homeostatic balance of C:N:P, resulting in an increase in the stoichiometric ratio C:N in shoots and C:P and N:P in roots, and in a decrease in the ratios C:Si, N:Si, P:Si and N:P in shoots and C:N, C:Si, N:Si and P:Si in roots (Fig. 2). Other studies have shown the benefits of Si in changing the homeostatic balance of C:N:P in different species. such as *Saccharum officinarum* L. [19, 39, 41–44], *Chenopodium quinoa* Willd. [34], *Sorghum bicolor* (L.) Moench [17, 33], and *Triticum aestivum* L. [35]. However, this is the first study to detect the role of Si in changing the stoichiometric ratio C:N:P in bean plants. These changes are expected, because Si modifies the concentration of carbon in plants, causing alterations in the rates of accumulation and use efficiency, especially under stress conditions [42, 54]. However, the extent of the changes induced by Si in beans is poorly understood. Si can reduce the changes caused by K deficiency in the C:N:P homeostatic balance.

We also evidence the benefit of Si in increasing the contents of N and P, that is, in the uptake of these nutrients by K-deficient bean plants (Fig. 4c, d, e and f). This is probably because Si increases the efficiency of NH_4_^+^ transporters (OsAMT) and the expression of NO_3_ transporters (BnaNTR2.1), in addition to improving the gene expression of inorganic phosphorus transporters (TaPHT1;1 andTaPHT1;2) [23]. Other studies on Si also support our results, showing the beneficial role of Si in improving N use efficiency [55, 56]. However, such studies did not investigate the implications of Si on the homeostatic balance of C, N, and P in plants.

The principal component analysis showed that the increase in the use efficiency of C, N, and P in bean plants is related to the presence of Si and the increase in the stoichiometric ratios C:Si, N:Si and P:Si (Fig. 7). Thus, the greater use efficiency of C, N and P modulated by Si makes clear the role of this beneficial element in increasing the ability of plants to use them in metabolism [12]. It consequently favors the conversion into dry mass of the shoots and roots of bean plants with potassium deficiency (Fig. 5g and h).

It becomes clear that a bean plant under K deficiency favors its ability to absorb more Si through the roots and accumulate less C in shoots because the plant in such a situation has a high C cost. Therefore, beans use a strategy that replaces Si for C, leading to a decrease in the stoichiometric ratio C:Si (Fig. 3a and b). This replacement of Si for C in the organic compounds of the cell wall has a low energy cost and may represent an energy saving ten to twenty times lower compared to the incorporation of C [57]. The energy balance resulting from the economy of replacing C and Si can be directed to the mechanisms of attenuation of nutritional stress caused by K deficiency. The latest evidence suggests that mono-silicic acid is complexed in the cell wall, forming Si–O-C bonds from the hydroxyl complexation between H_2_SiO_4_ and cisdiols [58].

Therefore, the beneficial effects of Si in mitigating a known K deficiency result not only from its role in restoring physiological activity impaired by nutritional deficiency [28, 59], as we show the underlying role of Si in the elementary stoichiometric homeostasis of C:N:P in bean plants. However, the hierarchical cluster analysis shows that despite the benefits of Si in attenuating the deleterious effects of K deficiency, such changes are still not enough to fully reverse the biological damage caused by deficiency of this macronutrient (Fig. 6a). This is because Si does not completely replace K functions in plants; it significantly mitigates the damage caused by K deficiency, improving plant growth, which has a great practical relevance.

In this scenario, our second hypothesis is true. It shows that the biological role of Si in attenuating K deficiency in bean plants occurs through the modification of the homeostatic balance of C:N:P, which increases the content and use efficiency of these nutrients, resulting in attenuation of biomass production losses.

Studies using Si in bean plants analyzed a predominance of different stresses [27, 60–62]. However, studies on this element in plants without stress are scarce, especially with legumes, as they are not Si-accumulating species but have the capacity to absorb this element.

### Silicon in the stoichiometry of C:N:P and nutritional efficiency in K-sufficient beans

The present study allows for an additional approach to the role of Si in the stoichiometry of C:N:P in bean plants without stress, that is, in plants with sufficient K. This helps to better understand why the use of this beneficial element can improve growth in plants in this situation.

The Si uptake capacity of bean plants with sufficient K was evidenced, as there was an increase in the concentration and content of the beneficial element (Fig. 1g, h, 4g, and h). This indicates a good application efficiency and there is no evidence of polymerization of the beneficial element in the solution. Si absorbed in plants predominates in the form of amorphous silica in the cell wall [58] and a small amount in the form of polysilicic acid [27].

The higher uptake of Si in bean plants with K sufficiency changed the homeostatic balance C:N:P, causing reductions in the C:N, C:P, C:Si, N:Si and P:Si ratios in shoots (Fig. 2a, c, 3a, c and e) and C:Si, N:Si and P:Si in roots (Fig. 3b, d and f). It also increased the N:P ratio in shoots (Fig. 2e) and C:N in roots (Fig. 2b).

Si can improve the efficiency of N use in roots and P in shoots and roots of plants (Fig. 5). The presence of this beneficial element contributes to the uptake and assimilation of these nutrients [23]. These results support the role of Si in alleviating N and P deficiency in plants by improving the uptake and accumulation of these nutrients [55, 63, 64]. Si caused changes in the homeostatic balance C:N:P in shoots and roots of bean plants. However, such stoichiometric modifications are not enough to improve the dry mass biosynthesis in plant shoots, restricting increases in dry mass only in roots (Fig. 5). This result may be related to the absence of an increase in the efficiency of N use in plant shoots (Fig. 5c), indicating that the modification of the stoichiometric ratios C:N and N:Si are not enough to improve the use of this admittedly important nutrient in plant physiological processes [20, 65, 66], which is vital for dry mass biosynthesis.

In addition, a greater effect of Si on root growth of a plant under no nutritional stress happens because the greatest benefit of Si in this organ arises from the replacement of C for Si in organic compounds, immobilizing Si in cell walls [67] with lower energy costs for biosynthesis [68]. Other authors reported the benefits of Si for root growth in plants without stress [69, 70], but these benefits did not reflect on a growth of bean shoots. Thus, our results reinforce the thesis that the greatest benefits of Si occur in plants under stress rather than in plants under no stress [71].

In this scenario, the third hypothesis is not true. The results, although they may indicate that Si generally potentiates the balance, the nutritional efficiency and the production of dry mass of roots, do not point to an increase in the production of shoot dry mass of common bean cultivated under K sufficiency. Therefore, a direct implication is that its use is not recommended for intensive agriculture without K restriction.

In general, our research makes it clear that the greatest benefit of Si in bean plants occurs under K deficiency. This finding has global implications given the low availability of this nutrient in crops, which occurs due to inadequate K fertilization caused, mainly, by an underdeveloped economy and scarcity of resources of soluble K [72], which are unevenly distributed in the world [73]. There is growing evidence that some agricultural systems have a limited availability of Si in soils [74], justifying the need to supply this element to obtain benefits in plants cultivated under K deficiency.

This research may pave the way for further studies with different species aiming to analyze the role of Si in the stoichiometric homeostasis of C, N and P, which are vital structural nutrients for plant metabolism, in order to better explain the optimal performance of crops, especially in soils with low potassium availability.

In conclusion, our study shows that K deficiency in bean plants causes biological damage to the homeostatic balance C:N:P, reducing the efficiency of nutrient use and causing losses in biomass production. Si is an alternative that can mitigate the damage caused by K deficiency, modifying the stoichiometric ratio C:N:P, increasing the use efficiency of nutrient, and reducing the loss of biomass production in bean plants. In plants with K sufficiency, Si also induces changes in the stoichiometric ratio C:N:P. However, these changes are not enough to increase shoot biomass, restricting gains only to the root system.

The future perspective is that the use of Si may be more intense in agriculture in underdeveloped economies with restriction of use of K. It may be a sustainable strategy to increase the productivity of bean crops and ensure food security.

## Methods

### Experimental conditions and design

The experiment was carried out in a controlled greenhouse at the Agricultural Production Science Department of the Faculty of Agrarian and Veterinary Sciences (FCA) of the São Paulo State University (UNESP), Jaboticabal, São Paulo, Brazil. During the experiment, the monitoring of climatic conditions was carried out using a digital thermohygrometer recording minimum temperature (19.5 ± 5 °C), maximum temperature (38.6 ± 7 °C), and relative humidity (32.8 ± 8%).

The factorial design was 2 × 2 randomized blocks, with two K supply conditions (deficiency at 0.2 mmol and sufficiency at 6 mmol) and two Si supply conditions (absence at 0.0 mmol and presence at 2 mmol), with five replications.

### Installation and conduction of experimental plots

Sowing was carried out in plastic seed trays in November 2017. The cultivar was BRS Estilo. Five days after seedling emergence, transplanting was carried out in 10-L polypropylene pots (0.44 × 0.19 × 0.14 m) containing sand, sand washed with water, and a solution of 0.5 mol HCl. The substrates of the experimental plots were washed weekly to eliminate excess salt using 700 mL of deionized water in each pot and pH adjusted to 5.5. The nutrient solution used in the experiment was that proposed by Hoagland and Arnon [75], that is, 10% concentration of the solution in the first week, 25% in the second week, and 50% from the third week until the end of the experiment.

The pH of the nutrient solution was kept between 5.5 and 6.5, correcting it with a solution of NaOH (1 mmol) and HCl (1 mmol). Si was supplied to the nutrient solution using SiNaK (1.8 mmol of Si and 0.2 mmol of K), balancing with K in treatments with no application of Si.

### Production of plant biomass

Twenty five days after seedling transplanting, the plants were collected and separated into shoots and root. They were washed in deionized water, neutral detergent solution (0.1%), HCl solution (0.3%), and deionized water. After washing, the samples were dried in a forced air oven until constant mass, determining the dry mass of shoots and roots.

### C, N, P, and Si concentrations

The determination of C was carried out by oxidation with K dichromate in acidic medium and titration of excess Cr^6+^. The concentration of N was determined by the Kjeldahl method of wet oxidation [76]. P concentration was determined by the nitric-perchloric digestion method and colorimetry (ammonium metavanadate method) [77]. Finally, Si was determined by alkaline digestion and reading was taken by colorimetry with ammonium molybdate [78].

### C:N:P:Si homeostatic balance

To determine stoichiometric ratios, the concentrations of elements in shoots and roots were used to estimate the stoichiometric ratios C:N, C:P, C:Si, N:P, N:Si, and P:Si.

### C:N:P:Si content and use efficiency

The contents of C, N, P, and Si were determined by multiplying dry mass by the concentration of the nutrient in shoots and roots. The use efficiency of C, N, and P was determined by the quadratic ratio of dry mass and nutrient content in shoots and roots [79].

### Statistical analysis of data

Shapiro–Wilk normality [80] and Levene homogeneity [81] tests were performed. Subsequently, an analysis of variance was performed (*p* < 0.05); when significant, a Tukey mean comparison test was performed (*p* < 0.05). Hierarchical cluster analysis was performed using the Euclidean distance coefficient, the group connection by single linkage, and principal component analysis (PCA) by covariance matrix. All data analysis procedures were performed using Python programming language (version 3.9.7; Python Software Foundation).

## Data Availability

The datasets generated and/or analyzed in this study are available from the corresponding author upon reasonable request.

## Abbreviations

C: Carbon
CUE: Carbon use efficiency
K_2_O: Potassium oxide
KCl: Potassium chloride
MSC: Dry mass of stems
MSF: dry mass of leaves
N: Nitrogen
NUE: Nitrogen use efficiency
P: Phosphor
PCA: Principal component analysis
PUE: Phosphor use efficiency
Si: Silicon

## References

[1] Sahoo A, Singh UK, Kumar MH, Samantaray S. Estimation of flood in a river basin through neural networks: A case study. 2021. p. 755–63.

[2] Liu K, Harrison MT, Yan H, Liu DL, Meinke H, Hoogenboom G (2023). Silver lining to a climate crisis in multiple prospects for alleviating crop waterlogging under future climates. Nat Commun.

[3] Hu W, Yang J, Meng Y, Wang Y,  Chen B, Zhao W (2015). Potassium application affects carbohydrate metabolism in the leaf subtending the cotton (Gossypium hirsutum L.) boll and its relationship with boll biomass. Field Crops Res.

[4] Ciceri D, Manning DAC, Allanore A (2015). Historical and technical developments of potassium resources. Sci Total Environ.

[5] Schlesinger WH (2021). Some thoughts on the biogeochemical cycling of potassium in terrestrial ecosystems. Biogeochemistry.

[6] Gerloff GC, Gabelman WH. Genetic basis of inorganic plant nutrition. In: Springer, editor. Encyclopedia of plant physiology. New series. 1983. p. 453–80.

[7] Zörb C, Senbayram M, Peiter E (2014). Potassium in agriculture – Status and perspectives. J Plant Physiol.

[8] Hu W, Coomer TD, Loka DA, Oosterhuis DM, Zhou Z (2017). Potassium deficiency affects the carbon-nitrogen balance in cotton leaves. Plant Physiol Biochem.

[9] Mahiwal S, Pandey GK (2022). Potassium: a vital nutrient mediating stress tolerance in plants. J Plant Biochem Biotechnol.

[10] Chen ZC, Liao H (2016). Organic acid anions: An effective defensive weapon for plants against aluminum toxicity and phosphorus deficiency in acidic soils. J Genet Genomics.

[11] Fang S, Yang H, Wei G, Shen T, Wan Z, Wang M (2022). Potassium application enhances drought tolerance in sesame by mitigating oxidative damage and regulating osmotic adjustment. Front Plant Sci.

[12] Prado RM (2021). Mineral nutrition of tropical plants.

[13] Liu Y, Song P, Zhang Y, Zhou D, Dong Q, Jia P (2023). Physiological mechanism of photosynthetic, nutrient, and yield responses of peanut cultivars with different tolerances under low K stress. Agronomy.

[14] Lopez G, Ahmadi SH, Amelung W, Athmann M, Ewert F, Gaiser T (2023). Nutrient deficiency effects on root architecture and root-to-shoot ratio in arable crops. Front Plant Sci.

[15] Ankit A, Singh A, Kumar S, Singh A (2023). Morphophysiological and transcriptome analysis reveal that reprogramming of metabolism, phytohormones and root development pathways governs the potassium (K+) deficiency response in two contrasting chickpea cultivars. Front Plant Sci.

[16] Marquez-Prieto AK, Palacio-marquez A, Sanchez E, Macias-Lopez BC, Perez-Álvarez S, Villalobos-Cano O (2022). Impact of the foliar application of potassium nanofertilizer on biomass, yield, nitrogen assimilation and photosynthetic activity in green beans. Not Bot Horti Agrobot Cluj Napoca.

[17] Hurtado AC, Chiconato DA, Prado R de M, Sousa Junior G da S, Olivera Viciedo D, Piccolo M de C. Silicon application induces changes C:N:P stoichiometry and enhances stoichiometric homeostasis of sorghum and sunflower plants under salt stress. Saudi J Biol Sci. 2020;27:3711–9. 10.1016/j.sjbs.2020.08.017 PMC7714968 33304182

[18] Teixeira GCM, Prado RM, Rocha AMS, Cássia PM (2020). Root- and foliar-applied silicon modifies C: N: P ratio and increases the nutritional efficiency of pre-sprouted sugarcane seedlings under water deficit. PLoS ONE.

[19] Oliveira Filho ASBP, Teixeira GCM, Cássia Piccolo M, Rocha AMS. Water deficit modifies C:N:P stoichiometry affecting sugarcane and energy cane yield and its relationships with silicon supply. Scientific Reports 2021 11:1. 2021;11:1–10. 10.1038/s41598-021-00441-0 PMC8536714 34686731

[20] Viciedo DO, Prado RM, Martínez CA, Habermann E, Piccolo M de C. Short-term warming and water stress affect Panicum maximum Jacq. stoichiometric homeostasis and biomass production. Science of The Total Environment. 2019;681:267–74. 10.1016/j.scitotenv.2019.05.108 31103664

[21] Das D, Ullah H, Tisarum R, Cha-um S, Datta A (2023). Morpho-physiological Responses of Tropical Rice to Potassium and Silicon Fertilization Under Water-Deficit Stress. J Soil Sci Plant Nutr.

[22] Zhang W, Xie Z, Lang D, Cui J, Zhang X (2017). Beneficial effects of silicon on abiotic stress tolerance in legumes. J Plant Nutr.

[23] Pavlovic J, Kostic L, Bosnic P, Kirkby EA, Nikolic M (2021). Interactions of silicon sith essential and beneficial elements in plants. Front Plant Sci.

[24] Patel M, Fatnani D, Parida AK (2022). Potassium deficiency stress tolerance in peanut (Arachis hypogaea) through ion homeostasis, activation of antioxidant defense, and metabolic dynamics: Alleviatory role of silicon supplementation. Plant Physiol Biochem.

[25] Andrade AF de, Bueno AM, Carvalho A dos S de, Lima ML de, Flores RA, Oliveira Abdala K de, et al. Innovative soluble silicon leaf source increase gas exchange, grain yield and economic viability in common bean. Silicon. 2022;14:3739–47.

[26] Calzada KP, Hurtado AC, Viciedo DO, Habermann E, Prado R de M, Oliveira R de, et al. Regulatory role of silicon on growth, potassium uptake, ionic homeostasis, proline accumulation, and antioxidant capacity of soybean plants under salt stress. J Plant Growth Regul. 2023;42:10921.

[27] Santos Sarah MM, Prado R de M, Souza Júnior JP, Teixeira GCM, Duarte JC dos S, Medeiros RLS. Silicon supplied via foliar application and root to attenuate potassium deficiency in common bean plants. Sci Rep. 2021;11:19690. 10.1038/s41598-021-99194-z PMC8490352 34608202

[28] Santos Sarah MM, Prado R de M, Teixeira GCM, Souza Júnior JP, Medeiros RLS, Barreto RF. Silicon supplied via roots or leaves relieves potassium deficiency in maize plants. Silicon. 2022;14:773–82.

[29] Barreto RF, Maier BR, Prado R de M, de Morais TCB, Felisberto G. Silicon attenuates potassium and sulfur deficiency by increasing nutrient use efficiency in basil plants. Sci Hortic. 2022;291:110616.

[30] Benslima W, Ellouzi H, Zorrig W, Abdelly C, Hafsi C. Beneficial effects of silicon on growth, nutrient dynamics, and antioxidative response in barley (Hordeum vulgare L.) plants under potassium deficiency. J Soil Sci Plant Nutr. 2022;22:2633–46.

[31] Benslima W, Zorrig W, Bagues M, Abdelly C, Hafsi C (2022). Silicon mitigates potassium deficiency in Hordeum vulgare by improving growth and photosynthetic activity but not through polyphenol accumulation and the related antioxidant potential. Plant Soil.

[32] Raven JA (1983). The transport and function of silicon in plants. Biol Rev.

[33] de Carvalho JS, Frazão JJ, de Mello PR, de Souza Júnior JP, Costa MG (2022). Silicon modifies C:N: P stoichiometry and improves the physiological efficiency and dry matter mass production of sorghum grown under nutritional sufficiency. Sci Rep.

[34] Lata-Tenesaca LF, Mello Prado R, Cássia Piccolo M, Silva DL, Silva JLF. Silicon modifies C:N:P stoichiometry, and increases nutrient use efficiency and productivity of quinoa. Scientific Reports 2021 11:1. 2021;11:1–9. 10.1038/s41598-021-89416-9 PMC8110966 33972664

[35] Neu S, Schaller J, Dudel EG (2017). Silicon availability modifies nutrient use efficiency and content, C:N:P stoichiometry, and productivity of winter wheat (Triticum aestivum L.). Sci Rep.

[36] Rocha JR, de Mello PR, de Cássia PM (2022). New outcomes on how silicon enables the cultivation of Panicum maximum in soil with water restriction. Sci Rep.

[37] Schaller J, Brackhage C, Gessner MO, Bäuker E, Gert DE (2012). Silicon supply modifies C:N: P stoichiometry and growth of Phragmites australis. Plant Biol (Stuttg).

[38] Wang Z, Lu J, Yang M, Yang H, Zhang Q (2015). Stoichiometric characteristics of carbon, nitrogen, and phosphorus in leaves of differently aged lucerne (Medicago sativa) stands. Front Plant Sci.

[39] Teixeira GCM, Prado R de M, Rocha AMS, Piccolo M de C. Silicon as a sustainable option to increase biomass with less water by inducing carbon:nitrogen:phosphorus stoichiometric homeostasis in sugarcane and energy cane. Front Plant Sci. 2022;13:826512. 10.3389/fpls.2022.826512 PMC9040072 35498639

[40] Oliveira Filho ASB, Prado R de M, Teixeira GCM, Rocha AMS, Souza Junior JP, Cássia Piccolo M, et al. Silicon attenuates the effects of water deficit in sugarcane by modifying physiological aspects and C:N:P stoichiometry and its use efficiency. Agric Water Manag. 2021;255:107006.

[41] Souza Júnior JP, Oliveira TL, Mello Prado R, Oliveira KR, Soares MB (2022). Analyzing the role of silicon in leaf C:N: P stoichiometry and its effects on nutritional efficiency and dry weight production in two sugarcane cultivars. J Soil Sci Plant Nutr.

[42] Costa MG, Prado R de M, Santos Sarah MM, Palaretti LF, Piccolo M de C, Souza Júnior JP. New approaches to the effects of Si on sugarcane ratoon under irrigation in Quartzipsamments, Eutrophic Red Oxisol, and Dystrophic Red Oxisol. BMC Plant Biol. 2023;23:51. 10.1186/s12870-023-04077-2 PMC9872329 36694112

[43] Costa MG, dos Santos Sarah MM, de Mello PR, Palaretti LF, de Cássia PM, de Souza Júnior JP (2022). Impact of Si on C, N, and P stoichiometric homeostasis favors nutrition and stem dry mass accumulation in sugarcane cultivated in tropical soils with different water regimes. Front Plant Sci.

[44] Frazão JJ, Prado R de M, Souza Júnior JP, Rossatto DR. Silicon changes C:N:P stoichiometry of sugarcane and its consequences for photosynthesis, biomass partitioning and plant growth. Scientific Reports 2020 10:1. 2020;10:1–10. 10.1038/s41598-020-69310-6 PMC7385645 32719349

[45] Kanai S, Ohkura K, Adu-Gyamfi JJ, Mohapatra PK, Nguyen NT, Saneoka H (2007). Depression of sink activity precedes the inhibition of biomass production in tomato plants subjected to potassium deficiency stress. J Exp Bot.

[46] Kanai S, Moghaieb RE, El-Shemy HA, Panigrahi R, Mohapatra PK, Ito J (2011). Potassium deficiency affects water status and photosynthetic rate of the vegetative sink in green house tomato prior to its effects on source activity. Plant Sci.

[47] White PJ, Karley AJ. Potassium. In: Hell R, Mendel RR, editors. Cell Biology of Metals and Nutrients. Springer. Berlin; 2010. p. 199–224.

[48] Gerardeaux E, Jordan-Meille L, Constantin J, Pellerin S, Dingkuhn M. Changes in plant morphology and dry matter partitioning caused by potassium deficiency in Gossypium hirsutum (L.). Environ Exp Bot. 2010;67:451–9.

[49] Imtiaz H, Mir AR, Corpas FJ, Hayat S (2023). Impact of potassium starvation on the uptake, transportation, photosynthesis, and abiotic stress tolerance. Plant Growth Regul.

[50] Champigny ML (1995). Integration of photosynthetic carbon and nitrogen metabolism in higher plants. Photosynth Res.

[51] Hafsi C, Debez A, Abdelly C (2014). Potassium deficiency in plants: effects and signaling cascades. Acta Physiol Plant.

[52] Mitani N (2005). Uptake system of silicon in different plant species. J Exp Bot.

[53] Minden V, Schaller J, Olde VH (2021). Plants increase silicon content as a response to nitrogen or phosphorus limitation: a case study with Holcus lanatus. Plant Soil.

[54] Kutasy E, Diósi G, Buday-Bódi E, Nagy PT, Melash AA, Forgács FZ (2023). Changes in plant and grain quality of qinter oat (Avena sativa L.) varieties in response to silicon and sulphur foliar fertilisation under abiotic stress conditions. Plants.

[55] Ma J, Ali S, Saleem MH, Mumtaz S, Yasin G, Ali B (2022). Short-term responses of Spinach (Spinacia oleracea L.) to the individual and combinatorial effects of Nitrogen, Phosphorus and Potassium and silicon in the soil contaminated by boron. Front Plant Sci.

[56] Parecido RJ, Soratto RP, Perdoná MJ, Gitari HI (2022). Foliar-applied silicon may enhance fruit ripening and increase yield and nitrogen use efficiency of Arabica coffee. Eur J Agron.

[57] Xia S, Song Z, van Zwieten L, Guo L, Yu C, Hartley IP (2020). Silicon accumulation controls carbon cycle in wetlands through modifying nutrients stoichiometry and lignin synthesis of Phragmites australis. Environ Exp Bot.

[58] Sheng H, Chen S (2020). Plant silicon-cell wall complexes: Identification, model of covalent bond formation and biofunction. Plant Physiol Biochem.

[59] Chen D, Cao B, Qi L, Yin L, Wang S, Deng X (2016). Silicon-moderated K-deficiency-induced leaf chlorosis by decreasing putrescine accumulation in sorghum. Ann Bot.

[60] Hussain S, Mumtaz M, Manzoor S, Shuxian L, Ahmed I, Skalicky M (2021). Foliar application of silicon improves growth of soybean by enhancing carbon metabolism under shading conditions. Plant Physiol Biochem.

[61] Portela GLF, Silva PRR, GirãoFilho JE, de Pádua LE, M, MeloJúnior LC de. Silicon as resistance inducer in to control black aphid Aphis craccivora Koch,  (1854). in Phaseolus lunatus lima beans. Arq Inst Biol (Sao Paulo).

[62] Moraes SRG, Pozza EA, Alves E, Pozza AAA, Carvalho JG, Lima PH (2006). Efeito de fontes de silício na incidência e na severidade da antracnose do feijoeiro. Fitopatol Bras.

[63] Araújo WBS, Teixeira GCM, de Mello PR, Rocha AMS (2022). Silicon mitigates nutritional stress of nitrogen, phosphorus, and calcium deficiency in two forages plants. Sci Rep.

[64] Silva JLF da, Prado R de M. Elucidating the action mechanisms of silicon in the mitigation of phosphorus deficiency and enhancement of its response in sorghum plants. J Plant Nutr. 2021;44:2572–82.

[65] Ferreira Barreto R, Schiavon Júnior AA, Maggio MA, de Mello PR (2017). Silicon alleviates ammonium toxicity in cauliflower and in broccoli. Sci Hortic.

[66] Campos CNS, Silva Júnior GB da, Prado R de M, David CHO de, Souza Junior JP de, Teodoro PE. Silicon mitigates ammonium toxicity in plants. Agron J. 2020;112:635–47.

[67] Schoelynck J, Bal K, Backx H, Okruszko T, Meire P, Struyf E (2010). Silica uptake in aquatic and wetland macrophytes: a strategic choice between silica, lignin and cellulose?. New Phytol.

[68] Schaller J, Brackhage C, Dudel EG (2012). Silicon availability changes structural carbon ratio and phenol content of grasses. Environ Exp Bot.

[69] Shamshiripour M, Motesharezadeh B, Rahmani HA, Alikhani HA, Etesami H (2021). Optimal concentrations of silicon enhance the growth of soybean (Glycine max L.) cultivars by improving nodulation, root system architecture, and soil biological properties. Silicon.

[70] Parecido RJ, Soratto RP, Guidorizzi FVC, Perdoná MJ, Gitari HI (2022). Soil application of silicon enhances initial growth and nitrogen use efficiency of Arabica coffee plants. J Plant Nutr.

[71] Souri Z, Khanna K, Karimi N, Ahmad P (2020). Silicon and plants: current knowledge and future prospects. J Plant Growth Regul.

[72] Mohammed SMO, Brandt K, Gray ND, White ML, Manning DAC (2014). Comparison of silicate minerals as sources of potassium for plant nutrition in sandy soil. Eur J Soil Sci.

[73] Manning DAC (2015). How will minerals feed the world in 2050?. Proceedings of the Geologists’ Association.

[74] Vandevenne F, Struyf E, Clymans W, Meire P (2012). Agricultural silica harvest: have humans created a new loop in the global silica cycle?. Front Ecol Environ.

[75] Hoagland DR, Arnon DI. The water-culture method for growing plants without soil. CAES. California; 1950.

[76] Tadesco MJ, Gianello C, Bissani CA, Bohnen H, Volkweiss SJ. Análises de solo, plantas e outro matériais. Porto Alegre; 1995.

[77] Carmo CAF do, Araújo WS de, Bernardi AC de C, Saldanha MFC. Métodos de análise de tecidos vegetais utilizados na Embrapa Solos. Rio de Janeiro; 2000.

[78] Korndorfer GH, Pereira HS, Nolla A (2004). Análise de silício: solo, planta e fertilizante.

[79] Siddiqi MY, Glass ADM (1981). Utilization index: A modified approach to the estimation and comparison of nutrient utilization efficiency in plants. J Plant Nutr.

[80] Royston P, Remark AS (1995). R94: A remark on algorithm AS 181: The W-test for normality. J Roy Stat Soc.

[81] Gastwirth JL, Gel YR, Miao W (2009). The impact of levene’s test of equality of variances on statistical theory and practice. Stat Sci.

